# Equitable inclusion of patients with cancer on the palliative care register: a systematic review in primary care

**DOI:** 10.3399/BJGP.2025.0004

**Published:** 2025-07-29

**Authors:** Rebecca Henderson, Sarah G Brearley, Maddy French, Natalie Ellis-Carr, Amy Gadoud

**Affiliations:** 1 Lancaster Medical School, Faculty of Health and Medicine, Lancaster University, Lancaster, UK; 2 Division of Health Research, Faculty of Health and Medicine, Lancaster University, Lancaster, UK; 3 Faculty of Biology, Medicine and Health, University of Manchester, Manchester, UK

**Keywords:** general practice, health inequalities, incurable cancer, palliative care, primary care, systematic review

## Abstract

**Background:**

GPs are instrumental to palliative care in the UK and most practices maintain a register of patients with palliative care needs. However, many people with incurable cancer who could benefit from palliative care are not included on this register, making this a potential source of end-of-life inequity.

**Aim:**

To identify barriers and facilitators to recognising the palliative care needs of patients with cancer in the UK and understand how these factors may have an impact on those vulnerable to end-of-life inequity.

**Design and setting:**

A mixed-methods systematic review.

**Method:**

Eight electronic databases (Alternative Medicine, CINAHL, Embase, Medline, PsycINFO, Scopus, SocINDEX, and Web of Science) and two preprint servers (medRXiv and Open Science Framework) were searched in November 2024. Qualitative, quantitative, and mixed-methods studies were included. Narrative synthesis was used to integrate study findings, with resulting barriers and facilitators mapped onto the COM-B model domains of capablity, opportunity, and motivation. The impact on equity was evaluated using the PROGRESS-Plus framework.

**Results:**

Searches returned 7190 citations, with 24 studies included in the review. Seven themes were identified, with barriers and facilitators mapped onto COM-B domains: conceptualisation of palliative care; navigating challenging conversations; healthcare organisation; patient help-seeking; time and resource constraints; perceptions shaping practice; and cognitive associations. There was insufficient evidence about the barriers and facilitators that may be having an impact on those vulnerable to end-of-life inequities.

**Conclusion:**

GPs experience many barriers to recognising palliative care needs. There is a need for greater understanding of the extent and nature of inequities in recognising such needs, to ensure strategies to increase recognition do not widen inequities.

## How this fits in

In the UK, GPs are incentivised to maintain a register of patients with palliative care needs. However, evidence suggests that many patients with incurable cancer who could benefit from palliative care are not included on the register, making it a potential source of inequity at the end of life. This review describes a theoretical approach to understanding the barriers and facilitators to the equitable recognition of the palliative care needs of people with incurable cancer by GPs in the UK primary care setting. Several high-level barriers and facilitators were identified, with many barriers reducing the opportunity for GPs to identify palliative care needs (such as loss of contact with patients and insufficient time and resources). However, findings demonstrate a paucity of research considering the identification of palliative care needs and factors previously associated with inequity at the end of life (such as socioeconomic characteristics, ethnic group, and age), with further research required to understand the identification of palliative care needs through an equity lens.

## Introduction

Palliative care is a multidisciplinary approach to care that aims to improve quality of life for people with life-limiting illnesses, addressing physical, psychosocial, and spiritual needs across the illness trajectory.^1,2^ GPs and the wider primary care team are instrumental providers of palliative care in the UK, with policy initiatives incentivising practices to maintain registers of patients with palliative care needs.^3,4^ There is no standardised approach to using palliative care registers, resulting in variance between practices. However, the aim is to help primary care teams monitor patients’ needs, develop personalised care plans, and coordinate their ongoing care through multidisciplinary meetings.^5^ These incentives are provided through frameworks such as the Quality and Outcomes Framework (QOF) in England and Northern Ireland^3,6^ and the Directed Enhanced Service (DES) in Scotland.^7^ Recent data suggest that 97%–100% of practices in England, Wales, and Northern Ireland have a palliative care register.^3,8,9^ Although 90.7% of practices in Scotland receive DES funding, it is unclear how many of these payments relate specifically to maintaining a palliative care register.^10^

Despite widespread engagement with these funding initiatives, evidence suggests that many people with palliative care needs are not on the register when they die.^11,12^ Unrecognised needs may result in inequities such as a lack of access to palliative care within the GP practice (such as out-of-hours services) and broader support (for example, financial assistance and access to specialist palliative care), as well as poorer end-of-life outcomes.^13–16^ People dying with non-cancer conditions experience inequities related to the identification of palliative care need. One study found that 78.8% of patients with chronic obstructive pulmonary disease or heart failure and 59.3% of patients with dementia were not included on the palliative care register when they died, compared with 38.1% of patients with cancer.^17^ These inequities have been attributed to less predictable illness trajectories that GPs describe as a barrier to discerning when patients require a palliative approach.^18,19^ Despite a higher proportion of patients with incurable cancer identified for palliative care, it is crucial to note that a considerable number of these patients are also not included on the register before they die.^12,17^ Thus, it is likely that inequities in the formal recognition of palliative care needs extend beyond issues related to diagnosis and prognosis, with further research required to understand why many patients with incurable cancer are not included on the register. Although previous research highlights many sociodemographic factors that contribute to end-of-life inequity (such as area deprivation, age, and ethnic group), these factors have primarily been explored within secondary and specialist palliative care, and not within the primary care setting.^20–22^ Accordingly, there is a need to identify the barriers and facilitators to inclusion of patients with incurable cancer on the palliative care register and consider factors that may contribute to inequitable inclusion.

The behaviour of healthcare professionals is central to the implementation of clinical guidelines and policy initiatives in practice.^23,24^ The COM-B model for behaviour change has been used to understand the factors that contribute to healthcare professionals’ behaviour, providing a theoretical lens to examine the implementation of recommendations.^25–30^ The model specifies that, for a behaviour to be enacted, people must have the capability, opportunity, and motivation to perform it:

- capability can be physical (for example, strength and stamina) or psychological (for example, cognition, knowledge, and skills);
- oppportunity can be physical (for example, time and resources) or social (for example, norms and social support); and
- motivation can be reflective (for example, decision making and intentions) or automatic (for example, emotions and habits)**.**

A limitation often attributed to behavioural models is their failure to account for wider social determinants, with resultant interventions found to widen inequities.^31,32^ Consequently, understanding the context in which the behaviours and associated outcomes occur is essential.^33^ Accordingly, this review uses the PROGRESS-Plus framework (recommended for equity-focused reviews) alongside the COM-B model to explicitly consider the following factors: place of residence, race and ethnicity, occupation, gender and sex, religion, education, socioeconomic status, social capital, and additional personal characteristics associated with discrimination (age, disability, and sexual orientation), illness-related factors, and relationships — many of which have previously been associated with end-of-life inequities.^34–36^

This review adopted the above frameworks as a theoretical lens to answer the question: ‘What are the barriers and facilitators to the equitable recognition of the need for a palliative care approach by GPs for patients with incurable cancer in the UK?’ The specific objectives were:

- to identify the barriers and facilitators to the inclusion of patients with incurable cancer on the palliative care register experienced by GPs; and
- to understand how barriers and facilitators may impact on patients vulnerable to end-of-life inequity.

## Method

This mixed-methods systematic review and narrative synthesis employed a convergent design to integrate findings from quantitative, qualitative, and mixed-methods research.^37,38^ The review was registered with the International Prospective Register for Systematic Reviews (reference: CRD42023427450) and is reported following the Preferred Reporting Items for Systematic Review and Meta-Analysis (PRISMA) checklist.^39^

### Eligibility criteria

Studies were eligible for inclusion when they met the criteria in Box 1.

**Box 1.** Eligibility criteria

**Population**	Studies that consider palliative and end-of-life care provided by GPs. This could be from the perspective of GPs, other healthcare professionals, patients, or carers/family members
**Patient group**	Adults (aged ≥18 years) with incurable cancer. This includes studies that consider adults with incurable cancer alongside those with non-malignant, life-limiting diagnoses
**Phenomena of interest**	The formal recognition of the need for a palliative care approach and/or inclusion on the palliative care register
**Context**	Primary care settings in the UK (including multinational studies that include the UK)
**Outcomes**	Factors that hinder or reduce the likelihood of identification/inclusion (barriers) or factors that enable or increase the likelihood of identification/inclusion (facilitators), either as described by the study authors or identified by the authors of this review
**Study design**	Any studies reporting primary research, including quantitative, qualitative, and mixed-methods designs, published since 2006 and in English

The review is context-dependent and focused on behaviour arising from current UK policy, which incentivises the maintenance of the palliative care register through initiatives such as the QOF.^3^ Although palliative care registers are utilised in countries outside of the UK, healthcare professional behaviour is embedded within the wider cultural, social, political, and economic context, necessitating a localised approach.^26^ Accordingly, inclusion was limited to research conducted in the UK and published between 2006 and 2024, in order to capture research published since the introduction of these incentives.^40^

### Search strategy

The search strategy was developed and piloted in collaboration with a subject librarian. It combined key terms and medical subject headings related to palliative care, incurable cancer, primary care, and the UK (see Supplementary Table S1). Searches were conducted on the following electronic databases, identifying articles published up to November 2024: Alternative Medicine, CINAHL, Embase, Medline, PsycINFO, Scopus, SocINDEX, and Web of Science. To identify unpublished research, two preprint servers were searched (medRXiv and Open Science Framework). Citations and references of included studies were also screened.

### Selection process

Citations from the database searches were imported into EndNote 20 and duplicates were removed. Titles and abstracts were screened against the eligibility criteria, with the full texts of all remaining studies retrieved and further assessed for eligibility. The reasons for exclusion were recorded. To minimise the risk of errors in applying the eligibility criteria and selecting studies for inclusion, 10% of the citations were independently screened by a second reviewer (the fourth author).

### Data extraction

A member of the research team (the first author) used a data extraction form to capture key study characteristics, a narrative summary of relevant findings, and equity characteristics (using the PROGRESS-Plus framework). Study findings were imported into ATLAS.ti for analysis, with quantitative results analysed qualitatively as recommended by the Joanna Briggs Institute for reviews of research with diverse methods.^38^ A copy of the data extraction form and extracted data are available on request from the authors.

### Quality appraisal

The study used the tool developed by Hawker *et al*,^41^ enabling the comparison of studies according to appropriate methodological standards. It produces a numerical score (maximum score of 36 points). Scores did not determine eligibility for inclusion in the review but were used to provide additional context during synthesis and reporting (see Supplementary Table S2).

### Data synthesis

Findings were integrated using the approach to narrative synthesis described by Popay *et al*.^37^ A preliminary synthesis was developed by analysing the findings of included studies inductively, using thematic analysis. Line-by-line descriptive codes were assigned to all findings related to the review objectives. Themes were developed to represent patterns identified across included studies and the resulting themes were applied to the theoretical framework. Relationships between identified barriers and facilitators were explored to develop a model of the recognition of palliative care need in primary care settings. To assess the robustness of the synthesis, the strength of the evidence was assessed using contextual information extracted from the studies, methodological limitations of the included studies, the adequacy of data supporting the finding, and the coherence of findings across the dataset.

## Results

**Figure 1.**
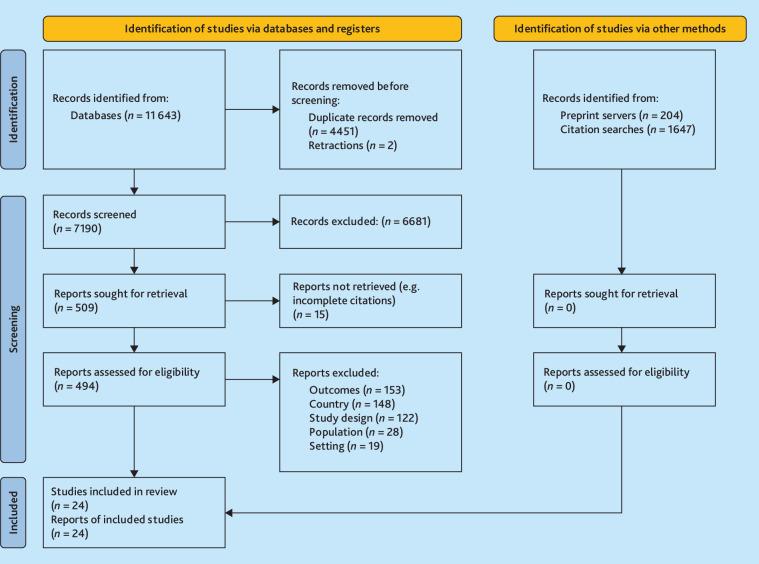
PRISMA flow diagram.

Searches returned 7190 unique citations, with 24 articles meeting the eligibility criteria (Figure 1).

### Study characteristics

The review includes quantitative,^12,42–44^ qualitative,^45–56^ and mixed-methods studies.^19,57–63^ Studies were conducted in various locations across the UK, including Scotland,^19,53,57–62^ Yorkshire,^43,46,48,49,56^ the Midlands,^44^ South West England,^42,52^ and South Wales,^47^ as well as those that collected data from multiple regions across the UK.^12,45,50,51,54,55,63^ One study was focused on England and New Zealand,^54^ but only findings related to England were synthesised. Supplementary Table S3 provides a summary of the included studies. Although most studies provided high-level data (for example, patient or practice demographics) for at least one PROGRESS-Plus factor,^12,19,42–50,52–54,56–63^ these were typically not explored in relation to the findings. Of the 24 included studies, 10 included PROGRESS-Plus factors when reporting their findings, which were illness-related factors,^43,46,49,50,52,60,63^ socioeconomic status,^42,52,56^ age,^12,52^ sex or gender,^52^ and place of residence.^42^ None of the studies reported data on patients’ race/ethnicity, religion, social capital, disability, or sexual orientation (see Supplementary Table S4).

### Quality of included studies

Almost all studies lacked evidence of rigour according to the appropriate methodological standards, including limited evaluation of bias or, for qualitative studies, evidence of reflexivity.^12,19,42–53,57–61^ Most of the included studies lacked sufficient contextual detail to assess the transferability of the findings, impeding the ability to make inferences about the impact on equity of identified barriers and facilitators.^19,44,45,47,48,50–54,57–59,61^ See Supplementary Table S2 for further details of the quality appraisal.

### Barriers and facilitators to recognising palliative care needs

Seven themes were developed: conceptualisation of palliative care; navigating challenging conversations; healthcare organisation; patient help-seeking; time and resource constraints; perceptions shaping practice; and cognitive associations between cancer and palliative care. The identified barriers and facilitators were analysed to understand their impact on capability, opportunity, and/or motivation. These findings are summarised in Box 2.

**Box 2.** Overview of themes

Theme	Barriers (COM-B impact)	Facilitators (COM-B impact)	Impact on equity (PROGRESS-Plus category)
Conceptualisation of palliative care	Palliative care conceptualised as end-of-life care **(reduces capability)**		Inclusion on the register was largely based on physical needs arising at the end of life. Thus, psychosocial and spiritual needs — or physical needs arising earlier in the illness trajectory — are less likely to trigger inclusion. **(Plus: illness-related factors)**
Navigating challenging conversations	Skills to discuss palliative care with patients, particularly given time and resource constraints **(reduces capability)** Beliefs about the consequences of discussing palliative care **(reduces motivation)** Insufficient time to hold sensitive discussions in appointments **(reduces opportunity)**.	Significant events, such as diagnosis or admission to hospital, to prompt conversations **(increases capability)**	
Healthcare organisation	Reactive processes for identifying patients with palliative care needs that rely on patient help-seeking behaviours **(reduces opportunity)** Inadequate communication between primary and secondary care **(reduces opportunity)** Loss of contact with patients receiving their cancer-related care in secondary care settings **(reduces opportunity)**	Proactive processes for identifying with palliative care needs **(increases opportunity)**	These barriers disproportionately have an impact on patients receiving their cancer-related care in oncology or haematology clinics and those with a longer illness duration **(Plus: illness-related factors)**
Patient and carer help-seeking	Patients not seeking help from the practice for their palliative care needs **(reduces opportunity)**		There was some evidence that patients are less likely to seek help for non-physical needs **(Plus: illness-related factors)**
Time and resource constraints	Increasing workloads and competing demands **(reduces opportunity and motivation)** Insufficient time during appointments **(reduces opportunity and motivation)**		
Perceptions shaping practice	Consequences of adding patients to the palliative care register perceived as negative, especially given time and resource constraints **(reduces motivation)** The provision of palliative care in general practice not perceived as different from usual care **(reduces motivation)** Perception that initiating conversations about palliative care may cause distress **(reduces motivation)**	Consequences of adding patients to the palliative care register perceived as positive **(increases motivation)** Clearly defined roles and responsibilities **(increases motivation)**	
Cognitive association between cancer and palliative care		Cognitive association between cancer and palliative care **(increases motivation)**	

COM-B = Capability, Opportunity, and Motivation — Behaviour model.

#### Conceptualisation of palliative care

GPs’ conceptualisation of palliative care was a barrier to identifying patients for inclusion on the palliative care register.^19,42,45,47,48,53–55,58–61^ This barrier typically arose when GPs’ definitions of palliative care lacked clarity or positioned it as synonymous with end-of-life care (care received in the final weeks of life) — even when acknowledging policy recommendations to integrate palliative care earlier in the illness trajectory.^19,45,47,53–55,58,60^ Conceptualising palliative care in this way meant inclusion on the palliative care register was primarily driven by physical needs and prognosis, rather than a holistic needs assessment including psychosocial and spiritual needs.^19,42,45,47,48,53–55,58–61^

#### Navigating challenging conversations

GPs’ skills and confidence in navigating challenging conversations had an impact on their capability and motivation to assess palliative care needs and identify patients for inclusion on the palliative care register.^19,42,45,53,55–62^ Many described their reluctance to use the word ‘palliative’ owing to its association with dying,^19,42,55,58–60^ with some delaying conversations or providing unrealistic information (for example, not disclosing to patients and carers what physical symptoms may arise as the illness progresses).^19,42,55^ One study indicated that GPs were more likely to discuss palliative care following a significant event, such as an admission to hospital or diagnosis,^61^ suggesting tangible events may facilitate communication with patients by increasing psychological capability. Furthermore, time and resource constraints reduce the opportunity for GPs to engage in conversations about palliative care. Both GPs and patients perceived appointments as rushed, with insufficient time for sensitive conversations about present needs and future care.^45,55–57^

#### Healthcare organisation

The way in which the healthcare system is organised for patients with incurable cancer affected GPs’ opportunity to identify patients for inclusion on the palliative care register.^19,42–51,53,58,60–63^ There was considerable variance in the proportion of patients included on the palliative care register, with primary care practices employing different processes to identify palliative care needs.^19,48,52,53,59,62^ Proactive processes (for example, routinely reviewing patients with cancer) facilitated inclusion by increasing opportunity and providing the time and resources required to identify patients.^19,47,48,53,58,60,61^ In contrast, reactive approaches that depend on patients actively seeking help for their palliative care needs are a barrier to recognising palliative care needs, reducing the opportunity for GPs to identify patients.^19,48^ Within the wider healthcare context, opportunity was further reduced by inadequate communication between settings.^42,45,48,50,60^ This was particularly pronounced for patients receiving most of their cancer-related care from secondary care settings, for whom GPs described loss of contact as an additional barrier.^43,46,49–51,63^ One study described an intervention that aimed to increase inclusion on the palliative care register using letters that specifically recommended patients be added to the register, thus improving communication between settings.^44^ Although the number of patients on the palliative care register increased, the data were insufficient to attribute this directly to the intervention.

#### Patient and carer help-seeking

Patients (and carers) did not always seek help for their palliative care needs, presenting an additional barrier to GPs that reduced their opportunity to identify patients for the palliative care register — especially when the practice relied on reactive processes.^12,19,42,43,45,46,49,53,55–58,63^ Barriers to help-seeking included a lack of knowledge about palliative care (including the scope and benefits of palliative care, misconceptions about the role of services, and where to seek support), beliefs about GPs’ availability and expertise, and their relationship with their GP.^43,45,46,53,56,57,63^ Lack of knowledge regarding sources of support appeared particularly pronounced for psychological, social, or economic needs, with help-seeking for psychological needs potentially further reduced by mental health stigma.^43,46,63^

Like GPs, many patients viewed palliative care as synonymous with end-of-life care, resulting in the perception that it was not currently relevant.^53,56,57^ Many patients appeared resistant to discuss palliative care owing to cultural attitudes towards death, associating palliative care with dying, and insufficient understanding about the focus on improving quality of life.^42,45,49,53,55–58^ Some patients said they preferred to ‘stay positive’, reinforcing the finding that conversations about palliative care were viewed negatively.^45,56,57^

#### Time and resource constraints

Time and resource constraints further reduced GPs’ opportunity to recognise palliative care needs, with GPs describing increasing workloads, competing demands for their time, and insufficient time in appointments as barriers to identifying patients with these needs.^45,54–61^ These barriers prevented GPs from adopting proactive processes and discussing palliative care with patients.^48,54,55,59^ Thus, the impact of time and resource constraints was not only limited to the direct reduction in opportunity, but also appeared to indirectly reduce both capability and motivation to engage in discussions about palliative care with patients.

#### Perceptions shaping practice

The way GPs perceived their role and the consequences of including patients on the palliative care register influenced their motivation to include patients on the register. Although GPs viewed maintaining the palliative care register as part of their role, there were differences in how they perceived this role that reduced motivation.^19,47,48,54^ Specifically, generalist palliative care was sometimes perceived as indistinct from the usual care provided by GPs, with ‘palliative care’ used to refer to specialist palliative care. In contrast, one study found that practices with proactive processes were more likely to view patients with advanced cancer as different from the general population, necessitating an alternative approach to care. This resulted in clear roles and responsibilities that increased GPs’ motivation to formally recognise palliative care needs.^48^

Additionally, beliefs about the consequences of adding patients to the palliative care register influenced GPs’ motivation.^19,45,53,57,59–61^ Positive beliefs (such as information sharing) increased motivation, whereas negative beliefs reduced motivation. The perceived negative consequences were closely related to time and resource constraints, with the perception that adding patients to the register increased workload and reduced care quality. This motivated some GPs to maintain a smaller list of included patients, in an attempt to ensure that high-quality palliative care was delivered to those perceived to have the greatest need.^48,55,57,60^

Motivation was also reduced by the perception that introducing the concept of palliative care may cause distress among patients, reinforcing the barriers associated with navigating challenging conversations, highlighting a relationship between motivation and capability.^19,42,57,59,60^

#### Cognitive association between cancer and palliative care

The final theme captures the cognitive association between incurable cancer and palliative care as a facilitator that increases GPs’ motivation by creating an automatic drive to include patients with incurable cancer on the palliative care register. This facilitator was evident in several studies, with patients who had cancer considerably more likely to be included on the register compared with patients with non-cancer conditions.^12,19,42,45,52,55,57,58,61,62^ The cognitive association arises from the historical context, with GPs thinking readily about patients with cancer as benefiting from palliative care.^33,40,48,49^ However, this still appeared to be contingent on prognosis, with no evidence that the association facilitates the recognition of needs arising earlier in the illness.^55^

### Inequity in the recognition of palliative care needs

None of the included studies specifically focused on equity, with only eight of the included studies providing insights on potential sources of inequity in the recognition of palliative care needs (see Supplementary Table S5). Seven studies suggested that some barriers may be more pronounced on the basis of illness-related factors, including type of needs and place of care.^43,46,49–51,53,63^ The conceptualisation of palliative care as synonymous with end-of-life care meant inclusion on the register was largely based on physical needs arising at the end of life, with psychosocial or spiritual needs less likely to trigger inclusion on the palliative care register, particularly when they arose earlier in the illness.^19,45,47,48,52–55,58–61^ There was also evidence that patients are less likely to seek help for non-physical needs, owing to a lack of awareness about where to seek support. The barriers associated with healthcare organisation disproportionally have an impact on patients receiving care in oncology and haematology clinics, who were less likely to seek cancer-related care from their GPs.^43,46,49–51,63^

Three studies reported Index of Multiple Deprivation (IMD, a composite measure used to capture area-based deprivation in England and Wales) in relation to the findings. Two provided the IMD decile alongside patient quotations,^42,56^ suggesting the associated barriers and facilitators were identified from patients living in areas of both high and low deprivation. Another found no association between IMD decile and inclusion on the palliative care register.^52^ However, this analysis used data from an opportunity sample of decedents from 20 general practices — with 86 practices contacted. The resulting dataset appears to be skewed towards more affluent areas, with 22% of the decedents in the most affluent decile compared with 3% in the most deprived decile. The low certainty of these findings mean it is not possible to make inferences about socioeconomic circumstances and inclusion on the palliative care register.

Two studies investigated the relationship between age and inclusion on the palliative care register. One found that older adults were less likely to be included,^12^ whereas the other found that older adults were more likely to have a palliative care record.^52^ The mechanisms underpinning these associations were unclear. One study found no association between gender and inclusion on the register.^52^ None of the studies considered place of residence, race or ethnicity, occupation, religion, education, social capital, disability, or sexual orientation in relation to inequity in the identification of palliative care needs.

## Discussion

### Summary

This review aimed to identify the barriers and facilitators to the equitable recognition of palliative care needs in primary care settings, with a focus on patients with cancer. Although the review highlights factors influencing the identification of palliative care needs, with more barriers than facilitators identified, the impact on equity of these, or factors that specifically have an impact on those vulnerable to end-of-life inequities, were rarely considered.

### Strengths and limitations

A strength of this review is the use of two well-established frameworks. COM-B is a recognised model for categorising the determinants of behaviour for implementing clinical guidelines^25,26^ and PROGRESS-Plus is recommended for reviews focused on inequity.^64^ The review was unable to fulfil its second objective to understand the impact on equity of barriers and facilitators to recognising palliative care needs, with these factors remaining poorly understood. Crucially, this means the findings of the review are insufficient to support equitable intervention development, as any resulting interventions would risk widening end-of-life inequities.^31,64,65^

### Comparison with existing literature

A key finding of this review was that insufficient time and resources are common barriers to GPs proactively assessing palliative care needs. Not only do these barriers directly reduce the opportunity for GPs to recognise palliative care needs, they also indirectly have an impact on behaviour through reduced capability (such as their ability to hold sensitive discussions) and reduced motivation (for example, GPs are motivated to manage their workloads to ensure high-quality care for those they perceive to have the greatest need). Consequently, GPs rely on reactive processes to identify patients for inclusion, dependent on patients actively seeking help for their palliative care needs. The wider literature suggests that these barriers may disproportionately have an impact on those at risk of end-of-life inequities. It is well established that populations living in deprived areas have greater health needs compared with those living in affluent areas.^66^ However, general practice resource allocation fails to account adequately for these differences.^67–69^ Practices in areas of high deprivation have fewer GPs and less funding per patient, with patients less likely to see a GP when they have an appointment.^70^ Although not specific to palliative care provision, these findings suggest that GPs providing care in deprived areas may have greater challenges related to their opportunity, capability, and motivation to identify palliative care needs.

The reliance on patient help-seeking presents additional barriers to recognising needs. Some of these are highlighted in this review, several barriers reducing the likelihood they would seek help from their GP, particularly for psychosocial needs. Although the impact on equity of this reliance on help-seeking was not adequately explored in this review, previous research implies a disproportionate impact on those vulnerable to inequity. For example, a lack of clarity about the role of general practice in facilitating psychosocial support may have a disproportionate impact on people living in poverty at the end of life, who are more likely to have complex psychosocial needs.^20^ Previous research has also identified additional barriers to help-seeking among marginalised communities. For example, a recent report on LGBTQ+ experiences of palliative care highlights a range of barriers to help-seeking, including anticipatory discrimination, past experiences, and heteronormative assumptions.^71^ Structural barriers to accessing health care, such as difficulties accessing translation, have also been found to have an impact on help-seeking among those with marginalised ethnicities.^72^ Consequently, reactive processes combined with a reliance on help-seeking may contribute to palliative care inequities in a myriad of ways, making these important topics for future research.

Although the review includes several studies that used routine data to examine inclusion on the palliative care register, none of these studies analysed the full range of equity-related characteristics available in routine data in relation to the inclusion on the palliative care register. Routine data are a valuable source for studying health inequity, particularly at the end of life,^73–75^ but challenges accessing the data required are widely reported.^76–78^ One study in this review noted unexpected restrictions that prevented access to data as planned in the protocol.^52,79^ Consequently, they recruited practices directly, which appeared to result in data skewed towards more affluent patients. These challenges highlight the need for greater collaboration between NHS trusts and researchers investigating palliative care using routine data, to ensure inequities do not remain invisible.

### Implications for research and practice

Although the review suggests a need to increase GPs’ capability by improving knowledge of palliative care, clarifying the scope of the palliative care register, and supporting them to manage difficult conversations, individual-level interventions (for example, targeting the capability of individual GPs) are likely to be insufficient to improve inclusion on the palliative care register. Organisational changes are required to increase GPs’ opportunity to identify patients with palliative care needs (for example, to introduce proactive processes and improve communication between settings). It is also essential to address the political context in which the behaviour occurs. Primary care is facing an unprecedented demand for services.^80–82^ Without policy-level changes to address unmanageable workloads and insufficient resources, it is unlikely that GPs will be able to identify all patients with palliative care needs, even when capable and motivated to do so, which may lead to inadequate symptom management, delayed access to services, increased hospital admissions, and reduced quality of life.^83–85^

Additionally, as this review highlights, most of the barriers related to capacity and motivation were linked to time and resource constraints. Although this review focuses on patients dying with cancer, challenges presented by the increasing demands on primary care services also have an impact on those with non-cancer conditions, who are less likely to be included on the register than those with cancer.^11,17^ Future research should engage with healthcare professionals, policymakers, commissioners, and patients to determine how these challenges may be overcome and, if necessary, explore alternative approaches to identification. It is vital that GPs and the wider primary care team are involved in these discussions, to ensure that their needs and priorities are considered.

Further research is required to investigate the recognition of palliative care needs through an equity lens, since interventions developed without this understanding may widen inequalities.^31^ This should include equity-focused primary research to investigate differences in GPs’ experience of identifying palliative care needs across the illness trajectory. Secondary research should further analyse routine data to investigate sociodemographic differences in inclusion on the register (such as, related to deprivation, age, and rurality), and the relationship between inclusion on the register and outcomes for patients vulnerable to end-of-life inequities. Although there are limitations to the use of routine data in this context, with many stratifiers of inequity not captured in primary care, it is a rich source of data that can overcome many of the challenges associated with palliative care research.^86^ A comprehensive, mixed-methods approach to future research on palliative care inequities in the primary care context can build a more nuanced understanding of the factors influencing identification and subsequent care, informing the development of interventions that account for the needs of individuals from marginalised communities.

In conclusion, this review highlights several barriers and facilitators that influence GPs’ inclusion of patients with incurable cancer on the palliative care register. Barriers associated with the conceptualisation of palliative care, communication (with patients and between settings), reactive processes, and time and resource constraints hinder GPs’ capability, motivation, and opportunity to identify patients for a palliative approach. Current pressures faced by primary care teams in the UK (for example, increasing workloads and competing demands) intensify these challenges. Although facilitators such as proactive processes and cognitive associations between cancer and palliative care were noted, it is uncertain whether these are sufficient given the considerable barriers identified. The review was unable to achieve its second objective, to understand factors contributing to inequity in inclusion on the register. Future research must consider the impact of differences in the identification of palliative care needs on equity, particularly those that have an impact on GPs’ opportunity for identifying patients to include on the palliative care register.

## References

[1] Lee GL, Ramaswamy A (2020). Physical, psychological, social, and spiritual aspects of end-of-life trajectory among patients with advanced cancer: a phenomenological inquiry. Death Stud.

[2] World Health Organization (2020). Palliative care. https://www.who.int/news-room/fact-sheets/detail/palliative-care.

[3] NHS England (2024). Quality and Outcomes Framework Guidance for 2023/24. https://www.england.nhs.uk/publication/quality-and-outcomes-framework-guidance-for-2023-24.

[4] Couchman E, Pocock L, Bowers B (2024). Reforming primary palliative care: a call to arms. Br J Gen Pract.

[5] National Institute for Health and Care Excellence (2019). End of life care for adults: service delivery. NG142.

[6] Department of Health (2023). Northern Ireland Quality and Outcomes Framework (QOF) information 2022/23. https://www.health-ni.gov.uk/news/northern-ireland-quality-and-outcomes-framework-qof-information-202223.

[7] NHS Scotland (2025). Claim for providing palliative care. https://www.nss.nhs.scot/medical-services/gp-practice-claims-and-mandates/claim-for-providing-palliative-care.

[8] Department of Health (2023). General practice Quality and Outcomes Framework: achievement and exceptions reporting statistics 2022/23. https://www.health-ni.gov.uk/sites/default/files/publications/health/qof-stats-ni-2022-23_0.pdf.

[9] Welsh Government (2024). Disease registers by local health board, cluster and GP practice. https://statswales.gov.wales/Catalogue/Health-and-Social-Care/NHS-Primary-and-Community-Activity/GMS-Contract/diseaseregisters-by-localhealthboard-cluster-gppractice.

[10] Public Health Scotland (2023). NHS payments to general practice: financial year 2022 to 2023. https://publichealthscotland.scot/publications/nhs-payments-to-general-practice/nhs-payments-to-general-practice-financial-year-2022-to-2023.

[11] Gadoud A, Kane E, Macleod U (2014). Palliative care among heart failure patients in primary care: a comparison to cancer patients using English family practice data. PLoS One.

[12] Gao W, Gulliford M, Morgan M, Higginson IJ (2020). Primary care service use by end-of-life cancer patients: a nationwide population-based cohort study in the United Kingdom. BMC Fam Pract.

[13] De Vleminck A, Houttekier D, Pardon K (2013). Barriers and facilitators for general practitioners to engage in advance care planning: a systematic review. Scand J Prim Health Care.

[14] Carey ML, Zucca AC, Freund MA (2019). Systematic review of barriers and enablers to the delivery of palliative care by primary care practitioners. Palliat Med.

[15] Mittmann N, Liu N, MacKinnon M (2020). Does early palliative identification improve the use of palliative care services?. PLoS One.

[16] Chapple A, Ziebland S, McPherson A, Summerton N (2004). Lung cancer patients’ perceptions of access to financial benefits: a qualitative study. Br J Gen Pract.

[17] Gadoud A, Kane E, Oliver SE (2024). Palliative care for non-cancer conditions in primary care: a time trend analysis in the UK (2009–2014). BMJ Support Palliat Care.

[18] Remawi BN, Gadoud A, Preston N (2023). The experiences of patients with advanced heart failure, family carers, and health professionals with palliative care services: a secondary reflexive thematic analysis of longitudinal interview data. BMC Palliat Care.

[19] Harrison N, Cavers D, Campbell C, Murray SA (2012). Are UK primary care teams formally identifying patients for palliative care before they die?. Br J Gen Pract.

[20] Reimer-Kirkham S, Stajduhar K, Pauly B (2016). Death is a social justice issue: perspectives on equity-informed palliative care. Adv Nurs Sci.

[21] French M, Keegan T, Anestis E (2021). Exploring socioeconomic inequities in access to palliative and end-of-life care in the UK: a narrative synthesis. BMC Palliat Care.

[22] Dixon J, King D, Matosevic T (2015). Equity in the provision of palliative care in the UK: review of evidence.

[23] Michie S (2014). Implementation science: understanding behaviour change and maintenance. BMC Health Serv Res.

[24] Grimshaw JM, Eccles MP, Lavis JN (2012). Knowledge translation of research findings. Implement Sci.

[25] Michie S, van Stralen MM, West R (2011). The behaviour change wheel: a new method for characterising and designing behaviour change interventions. Implement Sci.

[26] Michie S, Atkins L, West R (2014). The behaviour change wheel: a guide to designing interventions.

[27] McDonagh LK, Saunders JM, Cassell J (2018). Application of the COM-B model to barriers and facilitators to chlamydia testing in general practice for young people and primary care practitioners: a systematic review. Implement Sci.

[28] Rosário F, Santos MI, Angus K (2021). Factors influencing the implementation of screening and brief interventions for alcohol use in primary care practices: a systematic review using the COM-B system and theoretical domains framework. Implement Sci.

[29] Hart J, Byrne-Davis L, Maltinsky W, Bull E (2023). Training to change practice: behavioural science to develop effective health professional education.

[30] Heywood-Everett S, Henderson R, Webb C, Bland AR (2023). Psychosocial factors impacting community-based pressure ulcer prevention: a systematic review. Int J Nurs Stud.

[31] White M, Adams J, Heywood P, Babones SJ (2009). Social inequality and public health.

[32] Lorenc T, Petticrew M, Welch V, Tugwell P (2013). What types of interventions generate inequalities? Evidence from systematic reviews. J Epidemiol Community Health.

[33] Frohlich KL, Abel T (2014). Environmental justice and health practices: understanding how health inequities arise at the local level. Sociol Health Illn.

[34] Welch V, Petticrew M, Tugwell P (2012). PRISMA-equity 2012 extension: reporting guidelines for systematic reviews with a focus on health equity. PLoS Med.

[35] Tugwell P, Petticrew M, Kristjansson E (2010). Assessing equity in systematic reviews: realising the recommendations of the commission on social determinants of health. BMJ.

[36] Gomes B, Higginson IJ (2006). Factors influencing death at home in terminally ill patients with cancer: systematic review. BMJ.

[37] Popay J, Roberts H, Sowden A (2006). Guidance on the conduct of narrative synthesis in systematic reviews: a product from the ESRC methods programme.

[38] Lizarondo L, Stern C, Carrier J, Aromataris E, Lockwood C, Porritt K (2020). JBI manual for evidence synthesis.

[39] Page MJ, McKenzie JE, Bossuyt PM (2021). The PRISMA 2020 statement: an updated guideline for reporting systematic reviews. BMJ.

[40] NHS England (2007). Quality and Outcomes Framework — 2006–07. https://digital.nhs.uk/data-and-information/publications/statistical/quality-and-outcomes-framework-achievement-prevalence-and-exceptions-data/quality-and-outcomes-framework-2006-07.

[41] Hawker S, Payne S, Kerr C (2002). Appraising the evidence: reviewing disparate data systematically. Qual Health Res.

[42] Pocock LV, Wye L, French LRM, Purdy S (2019). Barriers to GPs identifying patients at the end-of-life and discussions about their care: a qualitative study. Fam Pract.

[43] Boele F, Harley C, Pini S (2024). Cancer as a chronic illness: support needs and experiences. BMJ Support Palliat Care.

[44] Lawrence E, Massey A, Whatley V (2024). Increasing the number of adults on a palliative care end‑of‑life register: a quality improvement project. Br J Healthc Manage.

[45] Mason B, Epiphaniou E, Nanton V (2013). Coordination of care for individuals with advanced progressive conditions: a multi-site ethnographic and serial interview study. Br J Gen Pract.

[46] Harley C, Pini S, Bartlett YK, Velikova G (2015). Defining chronic cancer: patient experiences and self-management needs. BMJ Support Palliat Care.

[47] Mitchell H, Noble S, Finlay I, Nelson A (2015). Defining the palliative care patient: its challenges and implications for service delivery. BMJ Support Palliat Care.

[48] Hackett J, Ziegler L, Godfrey M (2018). Primary palliative care team perspectives on coordinating and managing people with advanced cancer in the community: a qualitative study. BMC Fam Pract.

[49] McCaughan D, Roman E, Smith AG (2018). Palliative care specialists’ perceptions concerning referral of haematology patients to their services: findings from a qualitative study. BMC Palliat Care.

[50] McCaughan D, Roman E, Smith AG (2018). Determinants of hospital death in haematological cancers: findings from a qualitative study. BMJ Support Palliat Care.

[51] McCaughan D, Roman E, Smith AG (2019). Haematology nurses’ perspectives of their patients’ places of care and death: a UK qualitative interview study. Eur J Oncol Nurs.

[52] Pocock L, Morris R, French L, Purdy S (2024). Underutilisation of epaccs (electronic palliative care coordination systems) in end-of life-care: a cross-sectional study. BMJ Support Palliat Care.

[53] Hubbard G, Broadfoot K, Carolan C, van Woerden HC (2021). An exploratory qualitative study of computer screening to support decision-making about use of palliative care registers in primary care: GP think aloud and patient and carer interviews. J Prim Care Community Health.

[54] Gott M, Seymour J, Ingleton C (2012). ‘That’s part of everybody’s job’: the perspectives of health care staff in England and New Zealand on the meaning and remit of palliative care. Palliat Med.

[55] Wyatt K, Bastaki H, Davies N (2022). Delivering end-of-life care for patients with cancer at home: interviews exploring the views and experiences of general practitioners. Health Soc Care Community.

[56] Leach I, Mayland CR, Turner N, Mitchell S (2024). Understanding patient views and experiences of the identification of palliative care needs (IDENTI-PALL): a qualitative interview study. Br J Gen Pract.

[57] Mason B, Boyd K, Steyn J (2018). Computer screening for palliative care needs in primary care: a mixed-methods study. Br J Gen Pract.

[58] Zheng L, Finucane AM, Oxenham D (2013). How good is primary care at identifying patients who need palliative care? A mixed methods study. Eur J Palliat Care.

[59] Mason B, Boyd K, Murray SA (2015). Developing a computerised search to help UK general practices identify more patients for palliative care planning: a feasibility study. BMC Fam Pract.

[60] Mason B, Buckingham S, Finucane A (2015). Improving primary palliative care in Scotland: lessons from a mixed methods study. BMC Fam Pract.

[61] Finucane AM, Davydaitis D, Horseman Z (2020). Electronic care coordination systems for people with advanced progressive illness: a mixed-methods evaluation in Scottish primary care. Br J Gen Pract.

[62] Tapsfield J, Hall C, Lunan C (2019). Many people in Scotland now benefit from anticipatory care before they die: an after death analysis and interviews with general practitioners. BMJ Support Palliat Care.

[63] Reed E, Simmonds P, Haviland J, Corner J (2012). Quality of life and experience of care in women with metastatic breast cancer: a cross-sectional survey. J Pain Symptom Manage.

[64] O’Neill J, Tabish H, Welch V (2014). Applying an equity lens to interventions: using PROGRESS ensures consideration of socially stratifying factors to illuminate inequities in health. J Clin Epidemiol.

[65] Shoveller J, Viehbeck S, Di Ruggiero E (2016). A critical examination of representations of context within research on population health interventions. Crit Public Health.

[66] Marmot M (2020). Health equity in England: the Marmot review 10 years on. BMJ.

[67] Fisher R, Allen L, Malhotra AM (2022). Tackling the inverse care law: analysis of policies to improve general practice in deprived areas since 1990.

[68] Holdroyd I, Appel C, Massou E, Ford J (2024). Does adjusting the Carr-Hill formula, or total GP funding by deprivation data improve accuracy of predicting clinical need?. Future Healthc J.

[69] Levene LS, Baker R, Bankart J (2019). Socioeconomic deprivation scores as predictors of variations in NHS practice payments: a longitudinal study of English general practices 2013–2017. Br J Gen Pract.

[70] Fisher R, Dunn P, Asaria M, Thorlby R (2020). Level or not? Comparing general practice in areas of high and low socioeconomic deprivation in England. https://www.health.org.uk/sites/default/files/upload/publications/2020/LevelOrNot_Web1_0.pdf.

[71] Jerwood J, Allen G, Juffs H (2024). ‘It’s more than rainbows in receptions’ — working with LGBTQ+ people in palliative and end-of-life care.

[72] Jerwood J, Allen G (2023). No barriers here: for people excluded by identity, culture, ethnicity and race.

[73] Poots AJ, Green SA, Barnes R, Bell D (2012). Using routine geo-coded data to identify geographical heterogeneity to reduce disparities. https://dl.acm.org/doi/proceedings/10.1145/2452516.

[74] Leniz J, Higginson IJ, Stewart R, Sleeman KE (2019). Understanding which people with dementia are at risk of inappropriate care and avoidable transitions to hospital near the end-of-life: a retrospective cohort study. Age Ageing.

[75] Morisod K, Luta X, Marti J (2021). Measuring health equity in emergency care using routinely collected data: a systematic review. Health Equity.

[76] Lugg-Widger FV, Angel L, Cannings-John R (2018). Challenges in accessing routinely collected data from multiple providers in the UK for primary studies: managing the morass. Int J Popul Data Sci.

[77] Lensen S, Macnair A, Love SB (2020). Access to routinely collected health data for clinical trials — review of successful data requests to UK registries. Trials.

[78] Powell GA, Bonnett LJ, Tudur-Smith C (2017). Using routinely recorded data in the UK to assess outcomes in a randomised controlled trial: the trials of access. Trials.

[79] Pocock L, French L, Farr M (2020). Impact of electronic palliative care coordination systems (epaccs) on care at the end of life across multiple care sectors, in one clinical commissioning group area, in England: a realist evaluation protocol. BMJ Open.

[80] Health and Social Care Committee (2022). The future of general practice: fourth report of session 2022–23. https://committees.parliament.uk/publications/30383/documents/176291/default.

[81] McNamara P, Zubairi R (2021). The GP crisis: demonised and demoralised. Br J Gen Pract.

[82] Sinnott C, Dorban-Hall B, Dixon-Woods M (2023). Tackling the crisis in general practice. BMJ.

[83] Tavabie S, Ta Y, Stewart E (2024). Seeking excellence in end of life care UK (seecare UK): a UK multi-centred service evaluation. BMJ Support Palliat Care.

[84] Moghaddam N, Coxon H, Nabarro S (2016). Unmet care needs in people living with advanced cancer: a systematic review. Support Care Cancer.

[85] Cochrane A, Woods S, Dunne S, Gallagher P (2022). Unmet supportive care needs associated with quality of life for people with lung cancer: a systematic review of the evidence 2007–2020. Eur J Cancer Care.

[86] Davies JM, Gao W, Sleeman KE (2016). Using routine data to improve palliative and end of life care. BMJ Support Palliat Care.

